# Incidental advanced-stage Hodgkin lymphoma diagnosed at the time of radical prostatectomy for prostatic cancer: a case report and review of literature

**DOI:** 10.1186/1471-2407-14-613

**Published:** 2014-08-26

**Authors:** Antonio Di Meglio, Pier Vitale Nuzzo, Francesco Ricci, Bruno Spina, Francesco Boccardo

**Affiliations:** IRCCS San Martino University Hospital, IST National Cancer Research Institute, Academic Unit of Medical Oncology, Genoa, Italy; Department of Internal Medicine (DiMI), University of Genova School of Medicine, Genoa, Italy; IRCCS San Martino University Hospital, IST National Cancer Research Institute, Histopathology and Cytology Unit, Genoa, Italy

**Keywords:** Prostatic neoplasm, Radical prostatectomy, Hodgkin lymphoma, Hematologic neoplasm, Concurrent malignancies, CD44, Literature review

## Abstract

**Background:**

Pelvic lymph nodes removed during radical retropubic prostatectomy for prostatic cancer can be found on pathological examination to harbor various unexpected pathologies. Among these, hematologic neoplasms are not infrequent. Given their frequently indolent clinical course, such neoplasms would likely have remained undiagnosed and non-life threatening. Despite this, the case we are reporting describes a rare association between two aggressive neoplasms, and it will be helpful to clinicians who encounter similar combinations of pathologies.

**Case presentation:**

We report the challenging case of a 56-year-old, caucasian man in whom pathological assessment of pelvic lymph nodes removed during radical retropubic prostatectomy for a high-grade prostatic neoplasm revealed Hodgkin lymphoma, which was subsequently classified as stage IV. There are very few published reports of this combination of pathologies. This situation required a cautious and expert approach to delivering the most appropriate treatment with the most appropriate timing for both diseases.

**Conclusion:**

This report describes the multidisciplinary clinical approach we followed at our institution. We have also presented a review of published reports concerning the incidence, histologic type, and management of such concurrent malignancies.

## Background

Currently, radical retropubic prostatectomy (RRP) is considered the gold standard for local treatment of organ-confined prostate cancer (PCa) [1, 2]. Recognizing pelvic lymph node metastases from PCa during pre-operative assessment can be problematic. Because nodal involvement is often microscopic and therefore undetectable by using standard imaging techniques and dimensional and morphologic criteria, metastatic involvement of pelvic nodes can be overlooked preoperatively; only to be discovered unexpectedly by pathologists in the resected specimen [3, 4].

Several incidental findings, other than metastases from PCa, have been reported in pelvic lymph nodes evaluated at the time of RRP. These have included nodal metastases from malignancies arising in another primary site and non-metastatic disease arising directly from lymphoid tissue (i.e., various types of leukemia/lymphoma).

We describe the case of a patient who underwent surgery for a biopsy-proven high-grade PCa and had an incidental diagnosis of Hodgkin lymphoma (HL) involving pelvic lymph nodes. We then performed a systematic search of published reports concerning associations between PCa and hematologic malignancies (HM) discovered as a result of surgery for the PCa. Although several cases of concomitant HM and primary PCa have been reported, this association is uncommon; no guidelines for the management of such patients are thus far available. Moreover, the clinical significance and prognostic impact of these malignancies in the context of PCa remains unclear.

## Case presentation

### Case description

**Figure 1:**
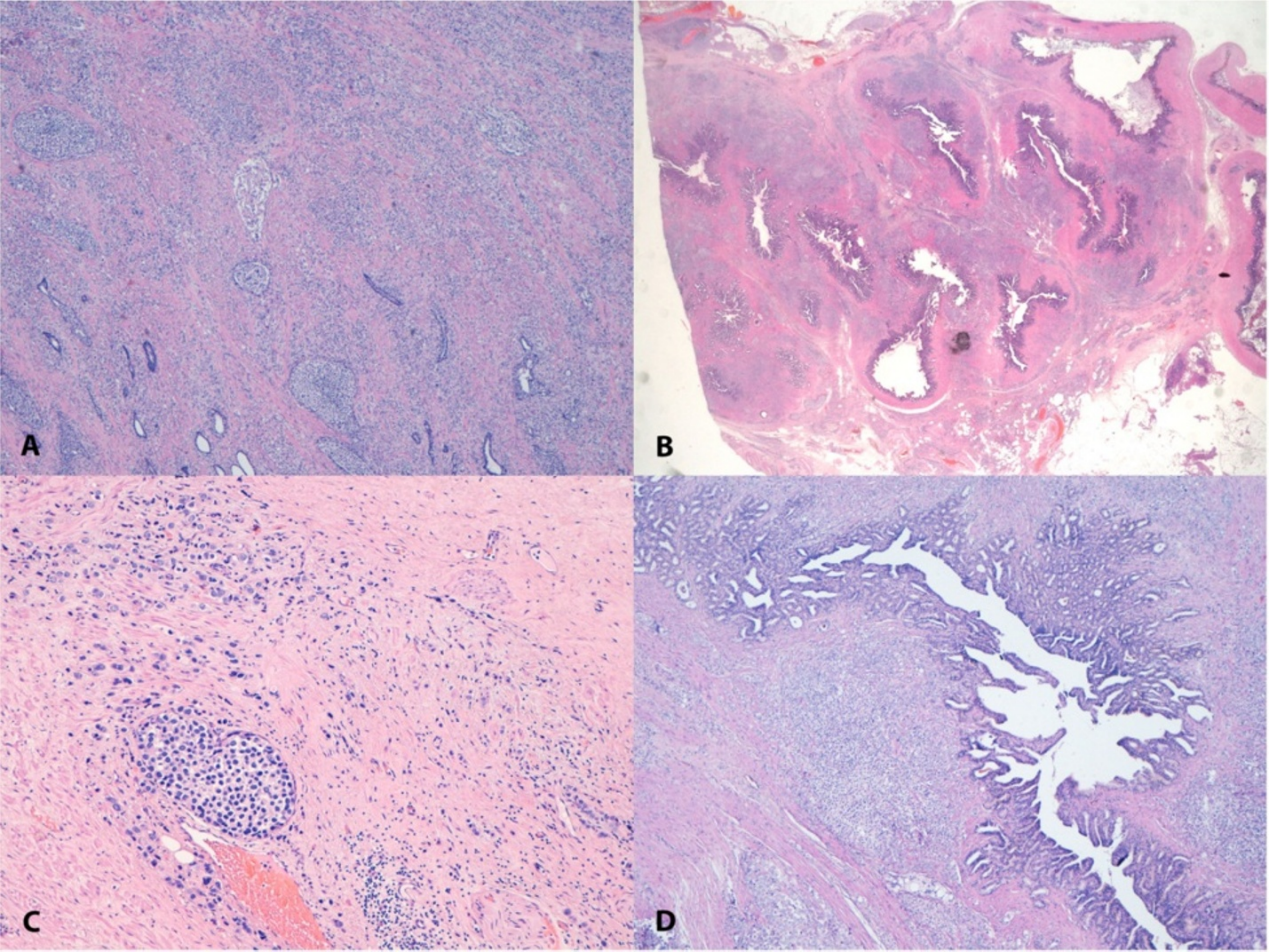
**Adenocarcinoma of the prostate, Gleason 10.** Hematoxylin and eosin stained photomicrographs (10x magnification) showing: **(A)** poorly differentiated adenocarcinoma of the prostate (Gleason score 5 + 5 = 10); **(B)** disease extension into seminal vesicles; **(C)** tumor vascular invasion; and **(D)** presence of multifocal embolic perineural tumor.

A 56-year-old man was referred to our unit after undergoing RRP and bilateral pelvic lymphadenectomy at another hospital. Pathological examination had confirmed the initial diagnosis of high-grade adenocarcinoma, Gleason score 10 (5 + 5), consistent with the findings on the biopsies performed preoperatively. Additionally, it had disclosed disease extension to both lobes of the gland, apex, and seminal vesicles, and focal involvement of the resection margins (Figure 1).

**Figure 2:**
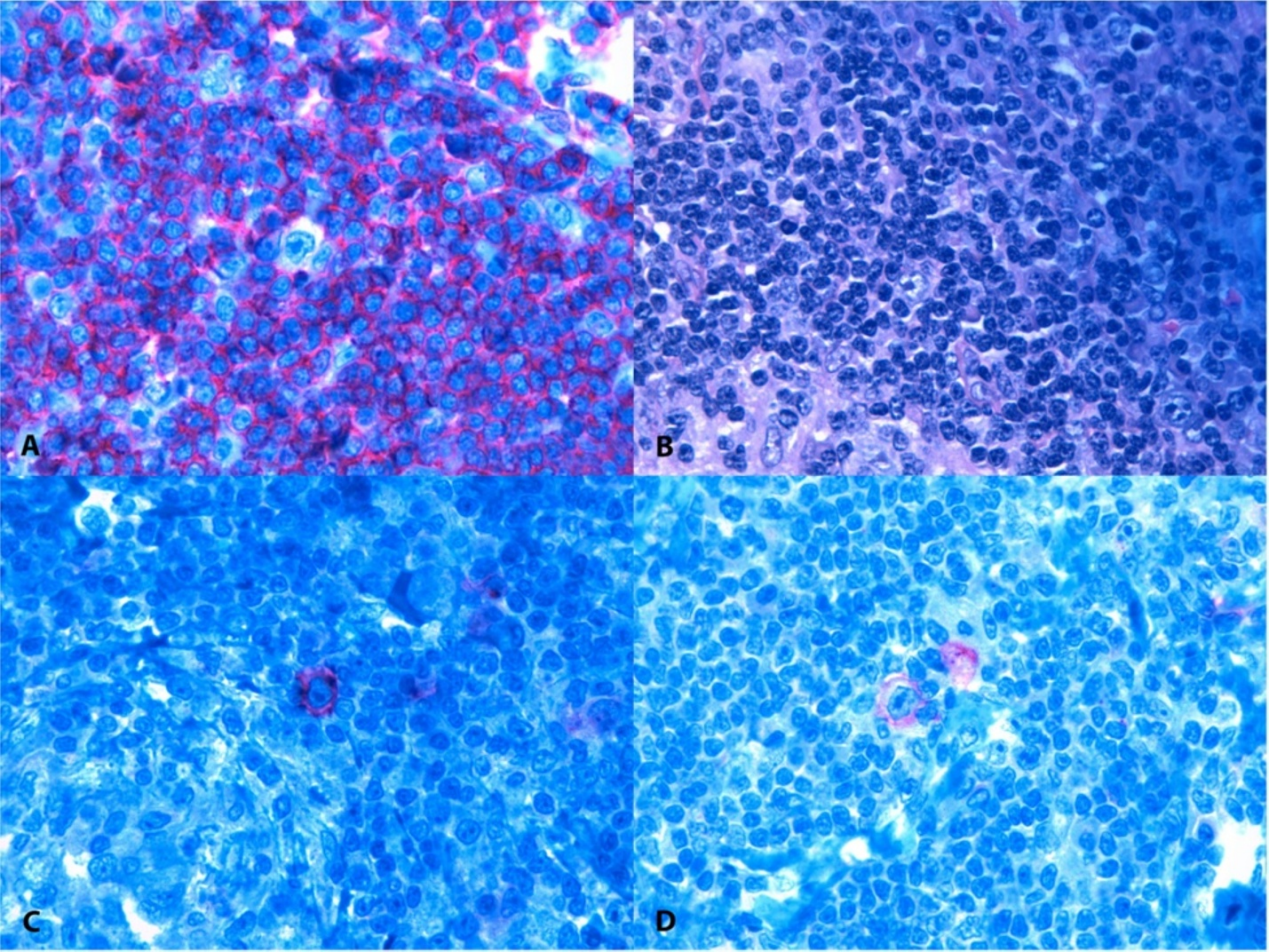
**Infiltration of Hodgkin lymphoma within lymph nodes.** Photomicrographs of **(A)** malignant Hodgkin and Reed-Sternberg cell showing **(B)** negative staining for CD45; **(C)** positive staining for CD30; and **(D)** positive staining for CD15.

None of the 30 lymph nodes removed in the procedure contained metastatic cells from the PCa. Rather and surprisingly, the larger lymph nodes were found to contain classic mixed cellularity HL. The malignant Hodgkin and Reed-Sternberg cells stained positive for cluster of differentiation (CD) 20, CD30, and CD15. Additionally, immunohistochemistry was negative for CD45, CD3, epithelial membrane antigen, and PAX5 (Figure 2).

When the patient was referred to our clinics 1 month postoperatively, his serum concentration of prostate-specific antigen (PSA) was 0.34 ng/mL (pre-surgical PSA had been 6.6 ng/mL).

**Figure 3:**
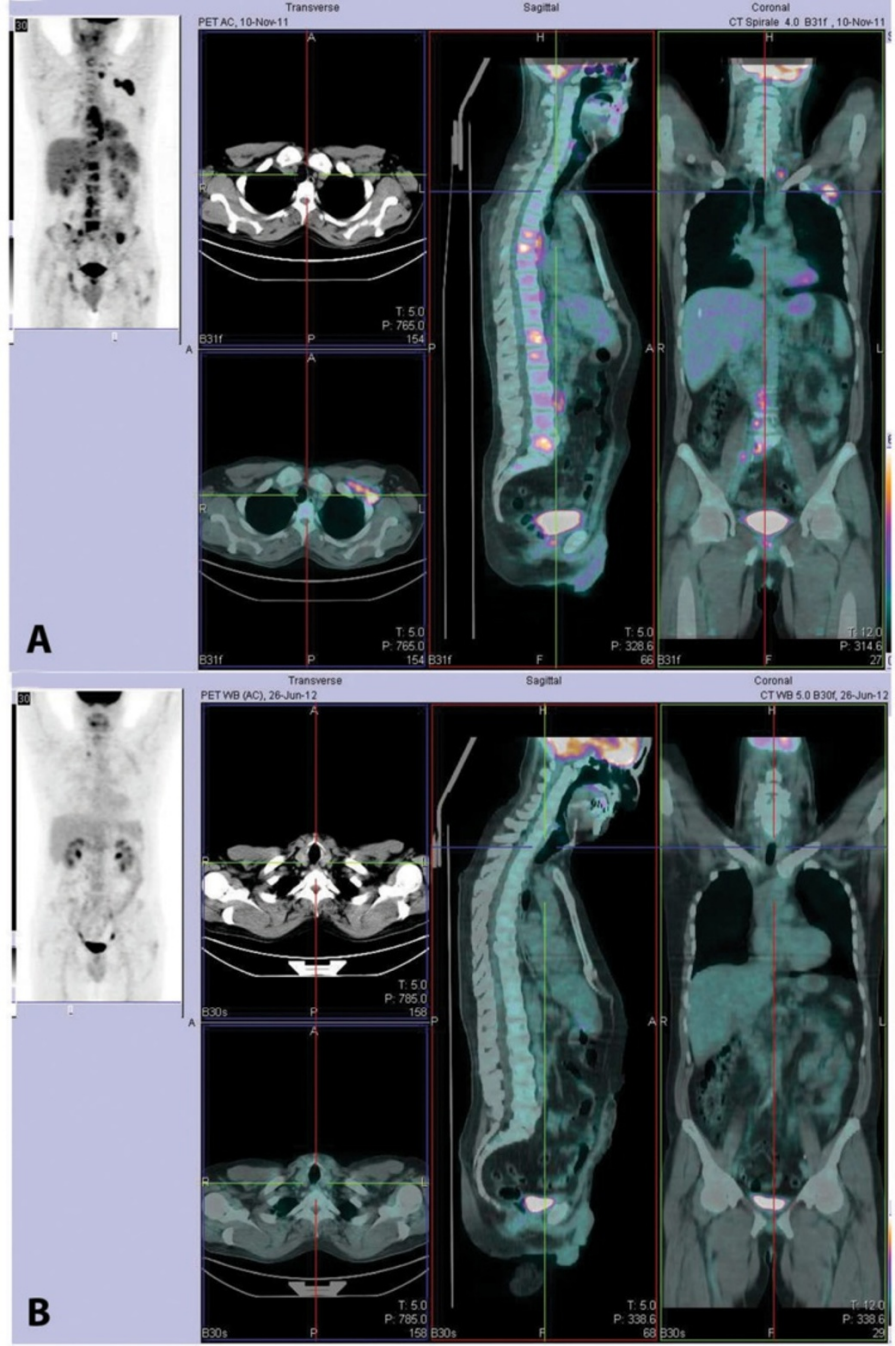
**FDG PET scan images before and after treatment. (A)** Staging FDG PET scan image showing nodal disease on both sides of the diaphragm with enhanced metabolic activity in the spleen and skeleton. **(B)** End of treatment: FDG PET image showing no residual disease.

A staging 18-fluoro-deoxyglucose positron emission tomography (FDG PET) scan showed nodal disease on both sides of the diaphragm with enhanced metabolic activity in the spleen and skeleton (Figure 3A). However, no tumor invasion was detected on bone marrow biopsy. A whole-body computed tomography (CT) scan confirmed axillary, mediastinal, celiac trunk, and retroperitoneal lymphadenopathies and failed to detect any bone lesions. Because the PET scan was positive at the bone level, his HL was classified as stage IV according to the Ann Arbor classification, even though bone involvement from PCa could not be completely excluded. The patient underwent front-line combination chemotherapy with the EBVD regimen (epirubicin 35 mg/m^2^; bleomycin 10 mg/m^2^; vinblastine 6 mg/m^2^; dacarbazine 375 mg/m^2^). A multidisciplinary team of experts, including hematologists and radiation oncologists, planned and concurred on this approach.

After three cycles of treatment, an interim evaluation with a FDG PET scan showed no FDG-avid tissue in the previously positive sites. These data were confirmed by a whole-body CT scan, which showed shrinkage of previously enlarged lymph nodes. Thus, there was evidence that the HL had responded well to chemotherapy; however, during this time the PSA concentration had further increased up to 0.96 ng/mL (PSA doubling time 1.92 months). Therefore, anti-androgen therapy with bicalutamide, 150 mg per day, was initiated. In addition to providing evidence of HL response to chemotherapy, the radiologic images also showed interstitial pneumonia, which was considered an adverse effect of bleomycin. Hence, three more cycles of chemotherapy without bleomycin and with the addition of 40 mg of prednisone daily on days 1–5 of each cycle were scheduled.

After six cycles of chemotherapy, a FDG PET scan showed no residual disease (Figure 3B); a whole-body CT scan confirmed complete disappearance of the lymphoma lesions and resolution of the interstitial pneumonia. PSA was undetectable in his serum. Nevertheless, because of the adverse prognostic features of his PCa; namely, the high Gleason score, invasion of seminal vesicles, positive surgical margins, and the increase in PSA concentrations postoperatively (before commencement of bicalutamide therapy), on completion of chemotherapy for HL, the patient was also submitted to pelvic irradiation (60 Gy were delivered in 30 fractions to the whole pelvis followed by an 18-Gy boost to the prostatic bed, which required the delivery of eight additional daily fractions). PSA continued to be undetectable in his serum up until completion of treatment and thereafter. Bicalutamide single-agent treatment is currently being continued and the patient is being rigorously followed up with serum PSA checks 3 monthly and whole body FDG PET/CT scans 6 monthly. At the time of this report, 30 months after this patient’s referral to our clinics, there is no evidence of either HL recurrence or of PCa progression (serum PSA remains undetectable).

### Discussion

We performed a systematic search of the PubMed database using the MeSH keywords "prostatic neoplasms", "prostatectomy", "lymphoma", and "hematologic neoplasms" and identified retrospective reviews of a total of over 19,000 specimens (most of which had been obtained from patients who had undergone RRP). We identified seven studies, designed *ad hoc* to assess the frequency and cause of incidental (non-metastatic) lymph node pathology discovered during RRP that had been performed between 1996 and 2007. The findings of these studies are summarized in Table 1.

**Table 1:** **Published reports of incidental concurrent findings during RRP and lymph node dissections for PCa**

Authors and year	Number of radical prostatectomies (Number of lymph node dissections performed)	Total number of concurrent hematolymphoid malignancies (Overall incidence)	Encountered Hematolymphoid malignancy	Treatment required for hematologic malignancy
Donohue *et al.* 1996 [5, 6]	225 (N.A.)	3 (1.2%)	Lymphoma NOS	N.A.
Terris *et al.* 1997 [7]	1092 (all patients)	13 (1.2%)	3 HL	Pelvic and abdominal external beam irradiation
			1 HCL	Aggressive tp NOS
			1 CLL	No tp
			6 SLL	No tp
Eisenberger *et al.* 1999 [8]	4319 (all patients)	10 (0.2%)	8 Lymphocytic lymphoma	Single-agent CT
			2 FL	N.A
Winstanley *et al.* 2002 [9]	1001 (854)	15 (1.8%)	2 SLL/CLL	N.A.
		3 neoplastic (0.003%)	1 FL	N.A.
			12 Non-neoplastic findings^1^	N.A.
Weir *et al.* 2003 [10]	6143 (all patients)	18 (0.3%)	18 SLL/CLL	N.A.
He *et al.* 2007 [11]	1500 (1150)	13 (1.13%)	9 SLL/CLL	N.A.
			3 MZL	N.A.
			1 MCL	Aggressive CT NOS
Chu *et al.* 2005 [12]	4831^3^ (N.A.)	29 (0.6%)	18 incidental cases	N.A.
			13 SLL/CLL	N.A.
			3 MZL	
			1 MCL	
			11 concurrent known lymphoma^2^	
			4 SLL/CLL	
			4 FL	
			2 MCL	
			1 DLBCL	
**Isolated case reports**	**Encountered Hematolymphoid malignancy**			**Treatment required for hematologic malignancy**
Carson H *et al.* 1996 [13]	1 SLL/CLL			N.A
Mydlo *et al.* 2001 [14]	1 lymphoma NOS			N.A
Drinis *et al.* 2009 [15]	1 SLL/CLL			No tp

Abbreviations used: *NOS* not otherwise specified, *tp* therapy, *N.A*. not available, *CT* chemotherapy, *HL* Hodgkin lymphom, *HCL* hairy cell leukemia, *CLL* chronic lymphocytic leukemia, *SLL* small lymphocytic lymphoma, *MZL* marginal zone lymphoma, *MCL* mantle cell lymphoma.

^1^Including sinus histiocytosis, non-caseating granulomas, foreign body reactions.

^2^Prostate and pelvic lymph nodes involved as part of a systemic disease.

^3^Specimens were from 3405 biopsies, 266 transurethral resections, and 1160 prostatectomies.

We also identified three case reports of patients who had been diagnosed with a second malignant hematologic neoplasm in addition to their PCa. These isolated cases are also listed in Table 1.

In the evaluated series, the overall incidence of HM harbored by pelvic lymph nodes removed in the course of RRP had a range from 0.003% [9] to 1.2% [7]. In the great majority of these cases, the diagnosis of a HM had not been suspected preoperatively.

Currently, contrast-enhanced CT scan along with MRI are the most commonly employed techniques for evaluating nodal disease pre-operatively in patients with PCa. These imaging techniques are usually reserved for patients with an intermediate or high risk of extra-prostatic and/or nodal disease dissemination [16, 17]. Evaluation of lymph node metastasis is one of the major goals of CT scanning in PCa staging. However, such evaluation is limited by false-positive results and the paucity of available techniques for identifying lymph node metastasis [18].

Moreover, unsuspected abnormalities, unrelated to the known primary PCa, can be revealed during the diagnostic/staging imaging workup. Miller *et al*. reported discovering a clinically significant coexistent disease by CT scan in 89/1330 PCa patients (6.7%) who were to undergo radiation therapy [19].

Elmi *et al*. retrospectively reviewed 355 initial staging abdominopelvic CT examinations in patients with PCa for incidental findings that were unrelated to their primary disease. These "incidentalomas" were classified as being of low, moderate, or high importance, depending on the type of medical or surgical management eventually required or on their potential to adversely affect health. Seventy-five potentially significant findings were noted in 73 patients (20.6% of all patients): most were renal masses; these were confirmed to be renal cell malignancies in seven patients (1.97% of all patients). Additionally, lymphadenopathies at sites unlikely to harbor PCa metastasis were noted in 18 cases, in four of whom histopathologic examination resulted in a diagnosis of lymphoma (1.12% of all patients) [20]. Enlarged lymph nodes were detected in 102 patients; only 18 of these were in sites uncommonly affected by PCa metastasis (mainly mesenteric). Accordingly, Coakley *et al*. suggested that a diagnosis of lymphoma should be considered in patients with PCa and imaging findings of mesenteric lymphadenopathies [21].

He *et al*. reported a <1% incidence of metastases from PCa in pelvic lymph nodes [11]. This rate of positivity is unusually low compared with major retrospectively assessed series reported by Roehl *et al*. and Daneshmand *et al*.: these authors cite an incidence of enlarged lymph nodes in typical PCa locations in the range of 5.8% [22] to 12.1% [23] in series of 3478 and 1972 patients, respectively, who had undergone RRP and lymph node dissection. However, Partin *et al*. have reported an even larger series of 5079 cases, considerably more than in either Roehl *et al*. or Daneshmand *et al*.’s series. These authors reported pathologically confirmed metastatic involvement by PCa of lymph nodes in 2% of the 5079 lymph node dissections performed [24].

Winstanley *et al*. have reported other findings apart from hematolymphoid pathology in enlarged pelvic nodes in patients undergoing RRP. Most such lymph nodes findings did not harbor neoplasms but were affected by other pathologies, including sinus histiocytosis, non-caseating granulomas, and foreign body reactions. Therefore, pathologists should be aware of these possibilities, to arrive at the correct diagnosis [9].

In the present case, our patient had not undergone any pre-surgical staging, probably because of his good general health and young age. In regard to age, Elmi *et al*. reported that the overall rate of incidental findings is not significantly different in patients aged <65 versus >65 years. However, they reported that patients aged more than 65 years have a higher rate of second neoplasms/synchronous malignancies than younger patients [20]. These findings are not relevant to our patient, who was aged less than 65 years.

Preexisting co-morbidities can influence treatment choices in patients with newly diagnosed PCa [25]. Though possible, incidental discovery of life-threatening conditions that may force clinicians to delay or modify the scheduled treatment for PCa is rare: imaging overuse can lead to over-diagnosis of subclinical conditions that would never become overt during a patient’s lifetime; this is a worldwide issue [26].

When discovered incidentally, HM are usually at an extremely early stage, have limited spread, and are asymptomatic [7, 11]. Although our patient was asymptomatic, he had stage IV HL involving lymph nodal stations on both sides of the diaphragm, as well as extra-nodal sites (spleen and skeleton). It is extremely rare to find such advanced disease incidentally. Of the cases identified by Eisenberger *et al*. in over 4000 procedures, none had diffuse and/or bulky disease [8].

Taking together, only six of 89 reported cases of incidentally discovered HM required an aggressive approach. Most reported patients with incidentally discovered HM had low-grade follicular non-Hodgkin lymphoma or small lymphocytic lymphoma/chronic lymphocytic leukemia. Considering the indolent nature of these conditions and the associated expected long-term survival, the decision to delay treatment until symptoms developed or disease-related complications occurred was made in the majority of patients reported [27].

No clinical management algorithms have yet been defined for synchronous occurrence of PCa and HL; the impact of such a double diagnosis on clinical outcome is unknown. Most authors suggest to treat the more aggressive condition first, thus improving the overall status of the patient and facilitating a better response of the second disease to therapy [28]. In our case, treatment decisions were jointly made by a panel of experts. Because HL appeared to be both the more aggressive of the two conditions and the disease in which cure was more likely to be achieved, combination chemotherapy with the EBVD regimen was initiated as soon as the patient had recovered from his surgery: complete remission of the disease was achieved within a few months.

To avoid any interference with the treatment for his lymphoma, our panel of experts decided to postpone pelvic radiotherapy, even though it was robustly indicated in view of the locally advanced stage of PCa and microscopic residual disease. The decision to postpone this treatment was supported by the prompt PSA response to the anti-androgen therapy initiated after the first two chemotherapy cycles. Though no randomized studies have demonstrated clear superiority for immediate treatment of biochemical recurrence with radiation or hormonal therapy, several retrospective studies have shown that anti-androgen therapy prolongs time to metastasis and probably PCa-specific survival [29, 30].

Despite the adverse histologic features and high Gleason score of our patient’s PCa, he has had no evidence of metastatic disease and no increase in PSA since completing pelvic radiotherapy.

As already mentioned, it is not clear yet whether the co-existence of a malignant lymphoma can alter *per se* the natural history of PCa.

Drinis *et al*. have raised the intriguing possibility that lymphomas could potentially protect against PCa progression. According to these authors, such protection could result from the over-expression of circulating transmembrane molecule CD44 in leukemia and lymphoma patients [15]. Some experimental studies support a tumor suppressor function of CD44 in lymphomas; silencing of CD44 expression may facilitate lymphoma genesis [31]. In contrast, circulating concentrations of this protein appear to be decreased in advanced and metastatic PCa, apparently contributing to tumor progression [32, 33].

Gao *et al*. have also suggested that CD44 is a "metastatic suppressor gene" in PCa. [34–36]. The biological role of CD44 might not be identical in all organs and tumors. In tissues that do not normally express CD44, its acquired expression probably correlates with an adverse outcome, the CD44 having growth- and metastasis-promoting actions [37–39].

**Figure 4:**
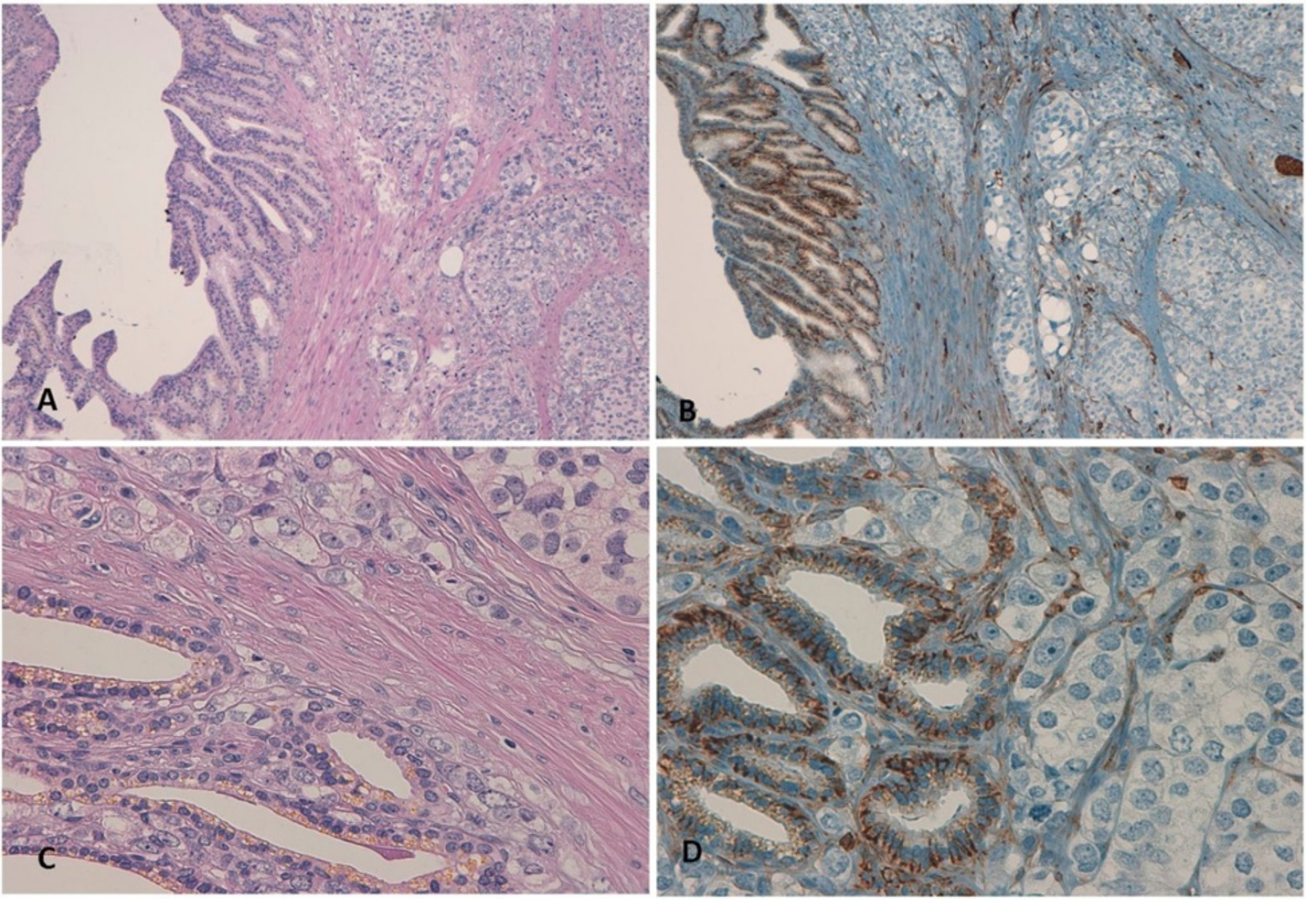
**Seminal vesicle infiltration by adenocarcinoma of the prostate (Hematoxylin/Eosin and CD44 staining). (A,C)** Hematoxylin and eosin stained photomicrographs showing left seminal vesicle infiltration by poorly differentiated adenocarcinoma of the prostate (10× and 40× magnification, respectively) **(B,D)** Photomicrographs showing positive CD44 staining of left seminal vesicle and negative staining CD44 staining of adenocarcinoma of the prostate (10× and 40× magnification, respectively).

In light of the above data, we performed CD44 immunostaining on the surgical specimens obtained from our patient during prostatectomy and lymph node dissection (Figure 4). The normal prostatic tissue stained positive for CD44, whereas the PCa tissue did not. The peri-tumoral stroma, seminal vesicles, and sites of perineural invasion were mildly CD44 positive. Lymph nodes involved by lymphoma also stained positive for CD44. These findings support the theory that CD44 is expressed by normal prostatic epithelium and that capacity for expression is lost during the alterations in structural differentiation that occur in the course of the transition to neoplastic tissue. Whether CD44 might be a prognostic marker indicating the malignant potential of neoplasm would be difficult to determine because a standard histologic scoring system that includes CD44 assay would be problematic because of the heterogeneity and available isoforms of this receptor [40]. Additonally, the interaction between CD44 expression by lymphoma cells and PCa cells remains unclear; further investigation is needed to assign a definite role to this transmembrane protein [41, 42].

## Conclusions

Apart from some speculations, we are not able to take a definitive stance about how the concurrent presence of a HM may affect or interfere with the natural history of PCa. What we can confidently state is that, in the present patient, the concurrent presence of a poor-risk PCa not only did not hamper treatment of the unexpected and newly diagnosed advanced-stage HL, but did not even hinder achievement of complete remission of the latter and long-term relapse-free survival.

## Consent

Written informed consent was obtained from the patient for publication of this case report and the accompanying images. A copy of the written consent is available for review by the Editor-in-Chief of this journal.

## Abbreviations

CD: Cluster of differentiation
FDG PET: 18-fluoro-deoxyglucose positron emission tomography
HL: Hodgkin lymphoma
HM: Hematologic malignancy
PCa: Prostatic carcinoma
PSA: Prostate-specific antigen
RRP: Radical retropubic prostatectomy.
